# Pollen-Associated Microbiome Correlates with Pollution Parameters and the Allergenicity of Pollen

**DOI:** 10.1371/journal.pone.0149545

**Published:** 2016-02-24

**Authors:** Andrea Obersteiner, Stefanie Gilles, Ulrike Frank, Isabelle Beck, Franziska Häring, Dietrich Ernst, Michael Rothballer, Anton Hartmann, Claudia Traidl-Hoffmann, Michael Schmid

**Affiliations:** 1 Research Unit Microbe-Plant Interactions, *Helmholtz Zentrum München*—German Research Centre for Environmental Health (GmbH), Neuherberg, Germany; 2 Institute of Environmental Medicine, UNIKA-T, *Technische Universität München*, Augsburg, Germany; 3 CK Care, Christine-Kühne-Center for Allergy Research and Education, Davos, Switzerland; 4 Institute of Biochemical Plant Pathology, *Helmholtz Zentrum München*–German Research Centre for Environmental Health (GmbH), Neuherberg, Germany; Oklahoma State University, UNITED STATES

## Abstract

Pollen allergies have been rapidly increasing over the last decades. Many allergenic proteins and non-allergenic adjuvant compounds of pollen are involved in the plant defense against environmental or microbial stress. The first aim of this study was to analyze and compare the colonizing microbes on allergenic pollen. The second aim was to investigate detectable correlations between pollen microbiota and parameters of air pollution or pollen allergenicity. To reach these aims, bacterial and fungal DNA was isolated from pollen samples of timothy grass (*Phleum pratense*, n = 20) and birch trees (*Betula pendula*, n = 55). With this isolated DNA, a terminal restriction fragment length polymorphism analysis was performed. One result was that the microbial diversity on birch tree and timothy grass pollen samples (Shannon/Simpson diversity indices) was partly significantly correlated to allergenicity parameters (Bet v 1/Phl p 5, pollen-associated lipid mediators). Furthermore, the microbial diversity on birch pollen samples was correlated to on-site air pollution (nitrogen dioxide (NO_2_), ammonia (NH_3_), and ozone (O_3_)). What is more, a significant negative correlation was observed between the microbial diversity on birch pollen and the measured NO_2_ concentrations on the corresponding trees. Our results showed that the microbial composition of pollen was correlated to environmental exposure parameters alongside with a differential expression of allergen and pollen-associated lipid mediators. This might translate into altered allergenicity of pollen due to environmental and microbial stress.

## Introduction

Over the last decades, incidences of allergic diseases have been increasing worldwide thus causing tremendous health problems and imposing considerable economic burden on the society [1]. A prime causative of allergies are airborne pollen grains from anemochorous plants (like trees, grasses or weeds) causing diseases like polinosis and hay-fever symptoms [2]. Sensitization rates to pollen allergens are estimated to range from 20% to 30% in Germany [3, 4]. The allergenic potential of pollen depends on the expression of immunogenic proteins–the allergens–as well as on non-allergenic molecules exerting pro-inflammatory or immune-modulatory effects [5]. Among these are NADPH oxidases, proteases, adenosine, or pollen-associated lipid mediators (PALMs) [6–8]. Some plant allergens, such as the birch pollen major allergen Bet v 1, belong to the pathogenesis-related (PR) family of proteins involved in host defense systems [9]. Specifically, Bet v 1 is proposed to confer protection from pathogens during pollen germination on the stigma [10]. Likewise, PALMs are assumed to play a role in the plant´s stress response to pathogens or heavy metal exposure [11, 12]. Plants constantly monitor the presence of microbes via specialized pattern recognition receptors. Analogous to immune cells in the animal kingdom, plant cells respond to pathogen associated molecular patterns, such as bacterial lipopolysaccharide, peptidoglycans, flagellin, or fungal chitin and β-glucan, by inducing specific host defense programs [13, 14]. Besides closure of stomata, host defense mechanisms can involve a burst of nitric oxide and reactive oxygen species production [15], which are both suggested to trigger PR-protein expression or synthesis of lipid mediators in tobacco cultures [16, 17]. Our previous data imply that those birch trees exposed to elevated ozone levels produce pollen grains with elevated levels of Bet v 1 and an altered PALMs composition, eventually leading to higher pro-inflammatory potential [18]. Furthermore, abiotic stress might alter a plant´s susceptibility to microbial pathogens [19]. In turn, pathogen-induced defense mechanisms might change the expression of allergens and adjuvant substances.

Most of the knowledge about plant-microbe interaction results from well characterized model organisms like *Arabidopsis thaliana* and studies of its rhizosphere. In contrast, little is known about microbial colonization of pollen and their influences on the allergen expression in pollen. In this study, we aimed to analyze and compare the microbial community on pollen from birch trees (*Betula pendula*) and timothy grass (*Phleum pratense*) which are both characterized by a high allergenic potential [2]. In addition, we aimed at investigating the correlations between pollen microbiota and parameters of air pollution and pollen allergenicity. For this reason, we collected pollen in urban and rural areas, analyzed their pollen-associated bacterial and fungal communities by performing a tRFLP analysis (terminal restricted fragment length polymorphism), and also did correlation (Spearman) and comparison analyses (PCoA, Cluster). Pollen obtained from birch and timothy grass showed a specific microbial pattern and, for both plant species, we detected an interrelationship between their microbial load and allergen content. Furthermore, we showed that the microbial diversity on birch pollen is correlated to environmental factors typical for urbanized areas. Our findings point out to previously unappreciated environment-plant-microbe interactions as being possible influencing factors of pollen allergenicity.

## Material and Methods

### Pollen Sampling and Preparation

In the flowering season of 2014, birch (*B*. *pendula*) catkins were sampled in the city and greater region of Augsburg, Germany. The number of pollen samples is equal to the number of birch trees (n = 20 urban, n = 35 rural). 100 catkins were sampled at each tree, at approximately 2 m height above the ground, and pollen were sieved from these catkins and pooled for each tree.

Timothy grass (*P*. *pratense*) spikes were collected from meadows in urban and suburban Munich as well as from rural meadows in Bavaria during the flowering season of 2014. For grass pollen samples, the number of pollen samples was equal to the number of meadows (n = 20). Pollen grains were extracted from the aforementioned birch catkins and grass spikes as it was described in Beck *et al*. [18]. Pollen grains were stored at -80°C. Aqueous pollen extracts of these samples were prepared as it was described in Gilles *et al*. [20].

### On-Site Measurements of Air Pollution Parameters

As to the air pollutant measurements, NO_2_, O_3_, NH_3_ were all measured on-site at each birch tree by means of passive samplers for the duration of eight weeks, beginning before flowering in march and spanning the whole pollen season as described in Beck *et al*. [18]. So there is one value for each measurement site/birch pollen sample (n = 55).

No on-site air pollutant measurements were performed at the grass pollen sampling-sites, only an urban index was calculated.

### Urbanization Index

The Urbanization Index (UI) was calculated on the basis of CORINE Land Cover data as described previously in Beck *et al*. [18]. Briefly, CORINE classifies land use in the area of the European Union into 44 categories. Areas with continuous urban fabric, discontinuous urban fabric, industrial or commercial units, road and rail networks and associated land, port areas, airports, mineral extraction sites, dump sites and construction sites are all classified as urban. The Urbanization Index shows the share of urban land use in the surrounding of 1km around the site. If UI = 1, the land use in the surrounding of the site is strictly urban, if UI = 0, the surrounding is completely of non-urban land use.

### Determination of Allergenicity Parameters

Levels of Bet v 1 and PALMs in aqueous pollen extracts were determined by ELISA as described in Beck *et al*. [18]. Group 5 grass pollen allergen (Phl p 5) was determined in aqueous grass pollen extracts by ELISA as described in Buters *et al*. [21].

### Determination of NADPH Oxidase Activity

NADPH oxidase activity measurement was adapted from Wang *et al*. [22]. In brief, pollen grains (1mg/assay) were hydrated in PBS and mixed with or without 2 mM nitroblue tetrazolium (NBT) (Sigma-Aldrich) and 1 mM nicotinamide adenine dinucleotide phosphate (reduced) (NADPH) (Roth) solved in pure water. The reaction (240 μl/assay) was started by adding the NBT. The pollen was afterwards incubated for one hour at 37°C under rotation. The obtained suspension was centrifuged and the NBT residual was removed by washing twice with 1 ml PBS. The produced formazan sediment was then resolved in 300 μl methanol under rotation for 30 minutes at room temperature and centrifuged again. Supernatants (200μl) were measured at 530 nm in a Tecan infinite M1000 PRO.

### tRFLP-Analysis of Bacterial Communities

Bacterial and fungal DNA from pollen was extracted with a Fast DNA Spin kit for soil (MP Biomedicals), according to the manufacturer’s instructions including an additional enzymatic lysis (10 μg of lysozyme, 30 min, 37°C). For the following steps each sample of extracted microbial DNA was divided in two parts, one for the determination of fungal pattern and one for the determination of bacterial pattern. In case of bacterial pattern firstly the 16S rRNA coding genes were amplified with a TopTaq DNA polymerase kit (Qiagen), a FAM-labeled forward primer 27f [23] and the reverse primer 907r [24]. Amplification was processed as follows: initial denaturation (94°C, 5 min), 30 cycles of denaturation (94°C, 45 sec), annealing (59°C, 45 sec), and elongation (72°C, 45 sec). A final extension at 72°C for five minutes completed the reaction. For the amplification of fungal ITS1-ITS2-region FAM-labeled primer ITS1F with the reverse primer ITS4 [25, 26] were used. After horizontal agarose gel-electrophoresis (120 V, 45 min), the amplicons were purified with a NucleoSpin Gel and PCR Clean up kit (Macherey & Nagel) according to the manufacturer’s instruction. For a rapid DNA digest, 16S rRNA gene products were restricted using the enzyme MspI (HpaII; 5´-C^CGG-3´; [27]) for 2h incubation at 37°C in recommended buffer. Amplified ITS-products, however, were restricted using the enzyme TaiI (MaeII; 5´-ACGT-3´) for 2h at 65°C in recommended buffer (Thermo Fisher Scientific). In pre-experiments regarding the efficiency and obtained fungal diversity the restriction enzymes TaiI performed best (Weikl F. 2015, pers. communication). After transferring the obtained fragments to HiDi Formamid (Applied Biosystems) including MapMarker ROX1000 (1:400; Bioventures), the denatured fragments were separated by an electrophoresis based on a capillary system on an automated DNA sequencer (ABI 3730, Applied Biosystems, Applera Deutschland GmbH, Darmstadt, Germany). The validation of data quality was done by using the online software package Peak Scanner Software v1.0 (Life technologies). True peaks (size < 50bp, height < 50 fluorescent units) were imported into t-REX (http://trex.biohpc.org/) for improving the data´s quality. Settings were adjusted according to Culman *et al*. [27] and Chowdhury *et al*. [28]. The data matrix of a bacterial pattern was first exported and then statistically analyzed in PAST ver. 2.17c [29]. In the following statistics of microbial pattern analysis the fragments (tRFs) were assumed to be derived from the same group of organisms in each sample.

### Exclusion of plastidic DNA

During amplification of bacterial 16S-rRNA coding genes, sequences of plastidic DNA from plant cells were co-amplified. For exclusion, the length of plastid-fragments was determined by tRFLP using the fluorescently labeled forward primer 27f [23], paired with the plastid-specific reverse primer CYA781r [30] for amplification. The obtained fragments were ligated and cloned by using the StrataClone cloning kit (Agilent technologies). For the colony PCR the forward primer T3 was used in combination with the reverse primer T7, both recommended in the manual of the StrataClone Cloning kit. Cloned fragments were sequenced at SequiServe GmbH (Vaterstetten, Munich, Germany). The hereby obtained sequences were aligned with the aligner tool implemented into the ARB software package [31]. For the case that a cloning procedure wants to be avoided, another well working opportunity for the exclusion of plastid DNA would be a 16S rRNA gene amplification with the primer pair 799f and 1492r. According to Chelius & Triplett 2001 [32] the 799f primer shows a different binding position in bacterial and plastid DNA. Hence, bands showing different length can be separated electrophoretically.

### Statistics

To compare the microbial composition of two groups the R-value and p-value of a one-way analysis of similarity (ANOSIM) was calculated as described in Clarke [33]. The ANOSIM R-value describes similarity-ranges from completely overlapping features (R = 0) to distinctly separated features (R = 1) of the compared communities [28]. For descriptive statistics, a principal coordinate analysis (PCoA, Bray-Curtis-Index) and a cluster-dendrogram (Bray-Curtis-Index) were performed. Additionally two indices referring to the diversity of the communities were calculated in Rstudio (Version 0.98.953, 2009–2013 RStudio, Inc.; 2014) [34]. To assign the alpha-diversity of the microbial composition the Simpson-Index 1-D (weighing high abundant fragments) and Shannon-Index H (weighing less abundant fragments) was assessed [35]. For comparison of numeric data, the significance level p was calculated by using the Man U Whitney-test in case of non-Gaussian distributed data. For Gaussian distributed data, the t-test for equal / unequal data was used to obtain the significance level. For correlations of measured pollution and allergen parameter to microbial diversity-indices, Spearman´s r_s_ and permutation p-value was calculated for non-Gaussian distributed samples [35]. For descriptive and univariate statistics of mean / median comparison as well as for correlations analyses, the statistical program PAST ver. 2.17c was used [29].

## Results

### Species Dependent Microbial Patterns on Pollen

**Fig 1:**
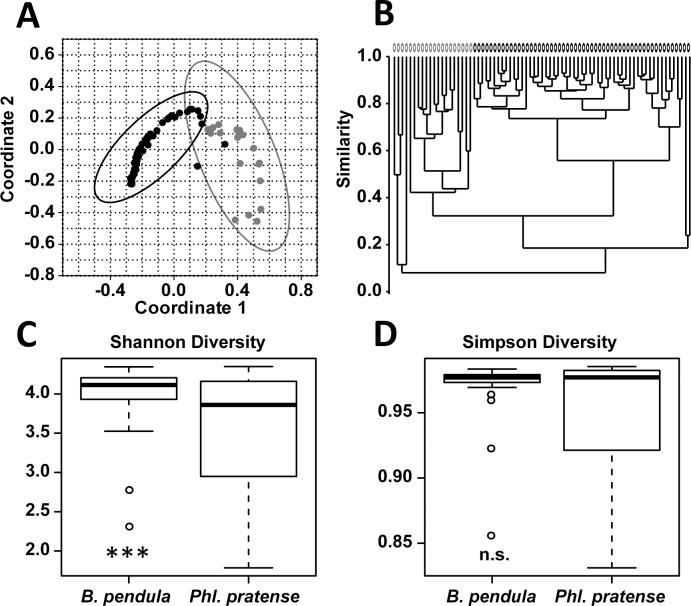
Comparison of bacterial pattern on pollen from birch and timothy grass. **A)** PCoA (Bray-Curtis-Index) of bacterial pattern on birch pollen (*Betula pendula*; n = 55; black) and timothy grass pollen (*Phleum pratense*; n = 20; grey) show hardly overlapping 95%-confidence intervals. The significant differences were confirmed by ANOSIM (R = 0.81, p = 0.0001). **B)** In the cluster dendrogramm (Bray-Curtis) pollen from both species are forming separated groups also attesting the significant difference. **C,D)** Boxplots of indices weighing species due to their extent of abundance in bacterial community on birch- (*Betula pendula*; n = 55) and timothy grass-pollen (*Phleum pratense*, n = 20). In comparison the two groups differs significantly due to low abundant species (Shannon-index, p = 0.009), but not significantly due to high abundant species of bacterial composition (Simpson-index, p = 0.78).

The bacterial pattern distribution of pollen from birch trees (n_birch_ = 55) and timothy grass (n_grass_ = 20) was illustrated in a PCoA (Principle Coordinate Analysis). The therein created 95%-confidence intervals were not overlapping. In an appending cluster dendrogram two separate clusters were formed (R = 0.81, p = 0.001). The diversity of the bacterial composition on birch tree and timothy grass pollen differed significantly due to low abundant bacterial species (Shannon index, p = 0.009) but not due to the dominant species (Simpson index, p = 0.78) (Fig 1).

**Fig 2:**
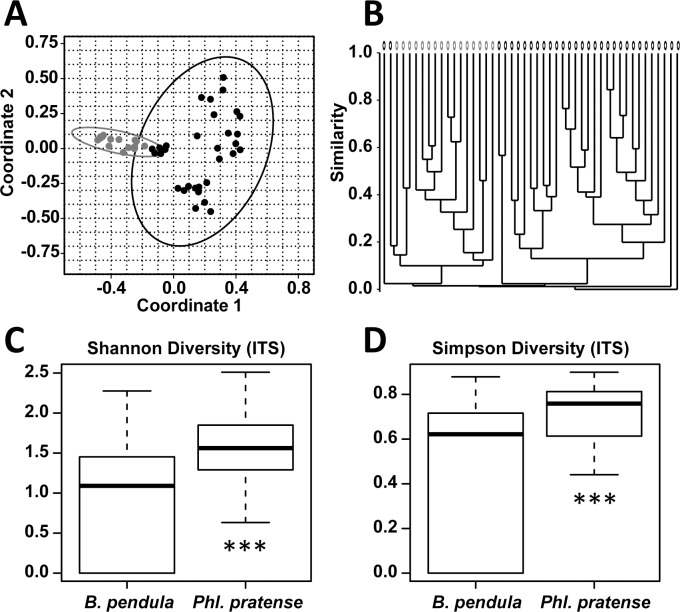
Comparison of fungal pattern on pollen from birch and timothy grass. **A)** PCoA (Bray-Curtis-Index) of fungal pattern on birch pollen (*Betula pendula*; n = 31; black) and timothy grass pollen (*Phleum pratense*; n = 16; grey) show hardly overlapping 95%-confidence. The significant differences were confirmed by ANOSIM (R = 0.51, p = 0.0001). **B)** The cluster dendrogramm (Bray-Curtis-Index) demonstrates the separation of the two pollen species due to fungal pattern. **C,D)** Boxplots of indices weighing species due to their extent of abundance in fungal community on birch- and timothy grass-pollen. In comparison the two groups differed significantly due to low abundant species (Shannon-index, p = 0.006), and also due to high abundant species of fungal composition (Simpson-index, p = 0.005).

When comparing the fungal community composition on pollen obtained from birch trees (n_birch_ = 31) and timothy grass (n_grass_ = 16) it differed significantly (R = 0.51; p = 0.0001) between the two of them. Similar to the bacterial community the 95%-confidence interval of the PCoA did not completely overlapped in this case, too. In the cluster dendrogram the separation of the two groups was less distinct than for bacterial patterns, but still was statistically significant. The diversity indices calculated, weighing either high abundant (Simpson-Index) or low abundant fungal species (Shannon-Index), showed that birch pollen had a significantly higher fungal diversity than timothy grass pollen (p__Shannon_ = 0.006, p__Simpson_ = 0.004) (Fig 2).

### Urban Sample Sites Show Different Bacterial Composition on Birch Pollen, but Not on Timothy Grass Pollen

**Fig 3:**
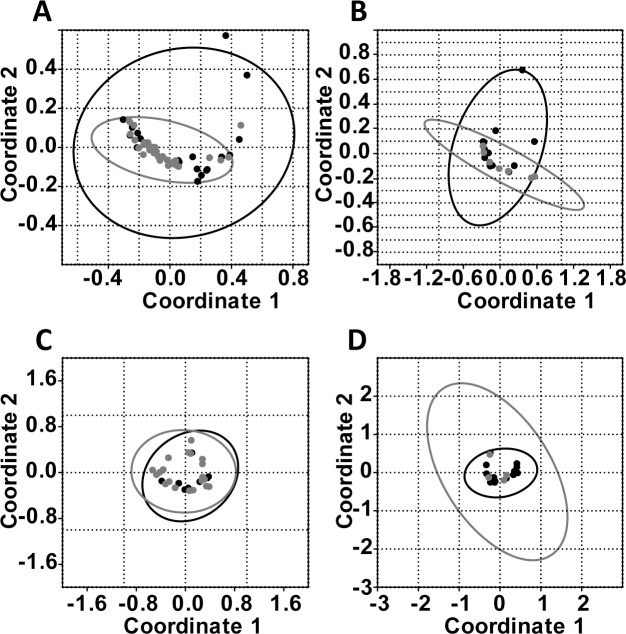
Comparison of bacterial and fungal pattern on pollen collected in rural and urban sample sites. PCoA performed to compare the microbial composition on pollen obtained from rural (grey) and urban areas (black). **A)** Bacterial composition on birch pollen. **B)** Bacterial composition on timothy grass pollen. **C)** Fungal composition on birch pollen. **D)** Fungal composition on timothy grass pollen.

The bacterial composition on the gathered pollen from urban sample sites partly show significant differences compared to the pollen collected in rural sample sites. Significant differences were detected for the bacterial pattern on birch pollen (n_urban_ = 20, n_rural_ = 35; R = 0.23, p = 0.003). Rural and urban timothy grass pollen samples (n_urban_ = 13, n_rural_ = 4) did not show statistically significant differences in bacterial composition (R = 0.02; p = 0.36). However, the fungal composition on birch tree (n_urban_ = 9, n_rural_ = 22) and timothy grass pollen (n_urban_ = 14, n_rural_ = 4) did not differ in urban or rural samples (R_birch_ = 0.04, p_birch_ = 0.62; R_grass_ = 0.09, p_grass_ = 0.69; Fig 3).

### Bacterial Diversity on Birch Pollen Was Correlated with NO_2_ Levels and Urbanization Index

Pollen was sampled from 55 birch trees and on-site air pollutant measurements were performed by means of passive samplers at each tree. Due to loss of passive samplers at a few sites, air pollutant measurements could be obtained for the following number of sampling sites: n(NO_2_) = 51, n(O_3_) = 52, n(NH_3_) = 49.

**Fig 4:**
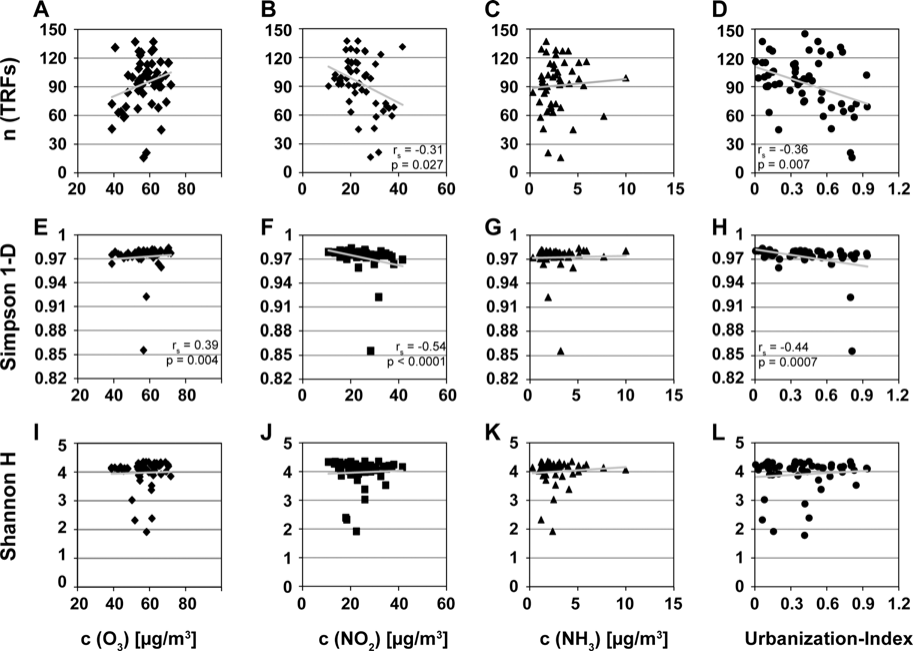
Spearman correlations of bacterial diversity on birch pollen to pollution parameter. Scatterplot including linear trend line, spearman correlation values rho and significance level p of diversity indices (Shannon H/Simpson 1-D) and parameter (number of fragments n(tRFs)) of bacterial pattern on birch pollen (*Betula pendula* 2014, n = 55) with pollution measurements. **A-D** Correlation of n(tRFs) and pollution parameters ozone O_3_ (r_s_ = 0.21, p = 0.13), nitrogendioxid NO_2_ (r_s_ = -0.31, p = 0.027), ammonia NH_3_ (r_s_ = 0.14, p = 0.34) and the urbanization-index (r_s_ = -0.36, p < 0.01). **E-H** Correlation of Simpson diversity Index 1-D and pollution parameters O_3_ (r_s_ = 0.39, p = 0.004), NO_2_ (r_s_ = -0.54, p < 0.0001), NH_3_ (r_s_ = 0.01, p = 0.94) and the urbanization-index (r_s_ = -0.44, p = 0.001). **I-L** Correlation of Shannon diversity Index H and pollution parameters O_3_ (r_s_ = 0.19, p = 0.17), NO_2_ (r_s_ = -0.14, p = 0.31), NH_3_ (r_s_ = 0.01, p = 0.89) and the urbanization-index (r_s_ = 0.06, p = 0.67).

Diversity indices of bacterial community on birch pollen were significantly correlated with pollution parameters of pollen sampling sites (Fig 4). Specifically, a significant negative correlation between the number of species (= total number of fragments; n(tRFs)) and ambient levels of NO_2_ (r_s_ = -0.31, p = 0.027; Fig 4B) was observed. In addition, a significant negative correlation between n(tRFs) and calculated UI (r_s_ = -0.36, p < 0.001; Fig 4D), but not to O_3_- (r_s_ = 0.21, p = 0.13; Fig 4A) or ammonia NH_3_-levels (r_s_ = 0.14, p = 0.34; Fig 4C) was observed. In contrast, the diversity of high abundant species (Simpson-Index) correlated significantly positive with O_3_-concentrations (r_s_ = 0.39, p = 0.0039; Fig 4E), but significantly negative to the calculated UI (r_s_ = -0.44, p < 0.001; Fig 4H) and NO_2_-level (r_s_ = -0.54, p < 0.0001; Fig 4F). There was no correlation of bacterial Simpson-diversity to NH_3_-levels (r_s_ = 0.01, p = 0.94; Fig 4G). The diversity of low abundant species (Shannon-Index) did not show significant correlations to any of the measured pollution parameters (r_s_ (O_3_) = 0.19, p(O_3_) = 0.16, Fig 3I; r_s_ (NO_2_) = 0.14, p(NO_2_) = 0.31, Fig 4J; r_s_ (NH_3_) = 0.01, p(NH_3_) = 0.89, Fig 4K; r_s_ (UI) = 0.06, p(UI) = 0.67, Fig 4L).

### Bacterial Diversity on Birch Pollen Correlated with Content of Bet v 1 and PALMs

**Fig 5:**
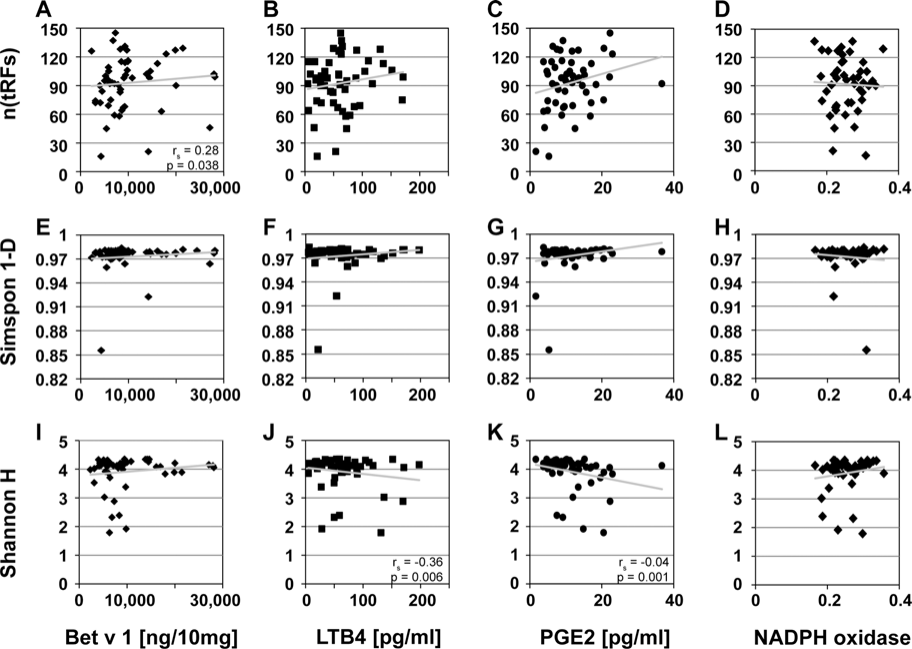
Spearman correlations of bacterial diversity on birch pollen to allergenicity parameter. Scatterplot including linear trend line, spearman correlation values rho and significance level p of diversity indices (Shannon H/Simpson 1-D) and parameter (number of fragments n(tRFs)) of bacterial pattern on birch pollen (*Betula pendula* 2014, n = 55) with allergen concentration in pollen. **A-D)** Correlation of n(tRFs) and allergenicity parameters Bet v 1 (r_s_ = 0.28, p = 0.038), PALM_LTB4_ (r_s_ = 0.14, p = 0.33), PALM_PGE2_ (r_s_ = 0.17, p = 0.22) and NADPH oxidase (r_s_ = -0.04, p = 0.79). **E-H)** Correlation of Simpson diversity Index 1-D and allergenicity parameters Bet v 1 (r_s_ = 0.25, p = 0.068), PALM_LTB4_ (r_s_ = 0.08, p = 0.53), PALM_PGE2_ (r_s_ = .12, p = 0.366) and NADPH oxidase (r_s_ = 0.2, p = 0.19). **I-L)** Correlation of Shannon diversity Index H and allergenicity parameters Bet v 1 (r_s_ = 0.14, p = 0.317), PALM_LTB4_ (r_s_ = -0.36, p = 0.006), PALM_PGE2_ (r_s_ = -0.04, p = 0.0015) and NADPH oxidase (r_s_ = 0.2, p = 0.19).

Diversity indices (Shannon, Simpson, n(tRFs)) calculated for the bacterial community on birch pollen were plotted against parameters defining the pollen´s allergenic potential (content of Bet v 1, PALMs, NADPH oxidase). Significant correlations were observed (Fig 5), specifically, the total number of different species (n(tRFs)) was positively correlated to the concentration of Bet v 1 (r_s_ = 0.28, p = 0.038; Fig 5A) but not to the concentration of PALM_LTB4_ (r_s_ = 0.14, p = 0.33; Fig 5B), PALM_PGE2_ (r_s_ = 0.17, p = 0.22; Fig 5C) or NADPH oxidase (r_s_ = -0.04, p = 0.79, Fig 5D). The diversity of high abundant species (Simpson-Index) showed no significant correlation with any of the measured parameters of the respective allergenic potential (r_s_ (Bet v 1) = 0.25, p(Bet v 1) = 0.068, Fig 5E; r_s_ (PALM_LTB4_) = 0.08, p(PALM_LTB4_) = 0.53, Fig 5F; r_s_ (PALM_PGE2_) = 0.12, p(PALM_PGE2_) = 0.366, Fig 5G; r_s_(NADPH oxidase) = 0.05, p(NADPH oxidase) = 0.75, Fig 5H). The diversity of low abundant species (Shannon-Index), however, was significantly negative correlated to the concentration of PALM_LTB4_ (r_s_ = -0.36, p = 0.006; Fig 5J) and PALM_PGE2_ (r_s_ = -0.04, p = 0.001; Fig 5K), despite a low r_s_-value. Shannon diversity did not correlate to the concentration of Bet v 1 (r_s_ = 0.14, p = 0.317; Fig 5I) or NADPH oxidase (r_s_ = 0.2, p = 0.19; Fig 5L).

### No Correlation between Fungal Diversity on Birch Pollen with Pollution or Allergenicity Parameters

In contrast to the bacterial patterns the composition of fungi on birch pollen grains were not significantly correlated to any of the pollution (NO_2_, O_3_, NH_3_) or allergenicity parameters (Bet v 1, PALM_PGE2_, PALM_LTB4_, NADPH oxidase) tested (S1 Table).

### Bacterial, but Not Fungal Diversity on Timothy Grass Pollen Correlated with the Content of PALM_PGE2_ and Urbanization Index

The statistical analysis of bacterial patterns obtained from timothy grass pollen resulted in a significantly negative correlation (r_s_ = -0.51, p = 0.039) between the diversity of high abundant species (Simpson-Index) and the PALM_PGE2_ content of pollen. Moreover, there was a significantly negative correlation between highly abundant species (Simpson-Index) (r_s_ = -0.57, p = 0.04) and the Urbanization Index (UI) and also between the total number of species (n(tRFs); r_s_ = -0.58, p = 0.04) and the Urbanization Index (UI). No further significant interrelationships were observed between bacterial diversity indices and parameters of allergenic potential (S2 Table). Similarly, no significant correlations were observed between fungal diversity on timothy grass pollen and allergenicity-related parameters or the Urbanization Index (S3 Table).

## Discussion

Plant-microbe interactions govern diverse physiological processes in plants such as growth regulation, nutrient supply, and host defense [36]. So far, however, only a few previous studies investigated the presence of microbes on plant pollen [37]. A more recent paper described the identification of gram-positive *Bacillus* species on timothy grass pollen. This paper also demonstrated that bacterial isolates can promote the maturation of human monocyte-derived dendritic cells [38]. To our knowledge, the present study is the first which systematically characterizes communities of bacteria and fungi on allergenic pollen grains and which also correlates the microbial colonization of pollen with parameters of environmental pollution and allergenicity. It has been known that anthropogenic pollutants, such as ozone and ultra-fine particulate matter, exert direct adverse effects on the human health [39, 40]. However, not only humans are exposed to air pollutants, but also the plants producing allergenic pollen. In recent years, it has become increasingly clear [5] that pollen allergenicity is not a constant parameter, but is readily influenced by biogenic and anthropogenic stress factors–among them are drought stress and such pollutions which cause stress to the allergenic plant [41, 42].

### Pollen Associated Microbiome Depends on Plant Species

In our present study, we firstly compared the microbial pattern on pollen grains of two different species, birch tree and timothy grass, and were thereby able to detect species-specific patterns due to bacterial and fungal colonization. The composition of microbial patterns as well as the extent of the microbial diversity revealed significant differences between birch tree and timothy grass pollen. Birch pollen showed a higher diversity in dominant bacterial species, but a lower fungal diversity than pollen obtained from timothy grass.

The microbial load of natural samples is influenced by several biotic factors (insect occurrence, contact to other plants) and abiotic factors (temperature, wind, humidity) which both characterize an environment [43, 44]. Birch catkins and timothy grass inflorescence are exposed to different environments caused e.g. by growth height or blooming period. Beside the presence of surface colonizing microbes in our microbial communities, also endophytes must be considered because of the mechanical and enzymatic cell-lysis during DNA-extraction. Amongst other bacteria, a strain of *Enterobacter cloacae* was previously identified in surface-sterilized pollen, roots, and shoots of several Pine species [45]. Translocational processes within the plants, like it is known for nutrients or heavy metals [46], may promote the existence of endophytic microbes in pollen. Other plant-specific parameters influencing the soil microbiome in the rhizosphere (e.g. root exudates altering pH-milieu or antimicrobial compounds [47]) would further impact the composition of endophytic microbes and might then result in diverse microbial pattern in pollen of different plant species. Another important factor influencing pollen-associated microbial colonization is the plant´s genotype. In experiments, mutants from *A*. *thaliana* which contain an altered cuticular wax biosynthesis presented a significantly different microbial composition on leaves compared to the plant´s wildtype [48]. Pollen of different plant species also shows a high phenotypical variability, thus influencing the occurrence of microbes, for instance a diverse structure of the exine [49] as well as various chemical and physical signals delivered by the exine in order to avoid inappropriate fertilization [50].

### Effect of Urbanization on the Pollen-Microbiome

Bacterial community patterns on timothy grass pollen did not differ between urban and rural sampling sites, whereas differences in bacterial patterns were observed between rural and urban birch pollen. The bacterial diversity (n(tRFs), Simpson-Index) correlated significantly negative with ambient NO_2_ levels and the calculated Urbanization Index. Ozone levels, in contrast, were positively correlated to the bacterial diversity (Simpson-Index), which was also visible by the trend of enhanced total species´ diversity (ntRFs).

A study analyzing the impact of O_3_ to soil microbiome in the rhizosphere showed that O_3_ influences bacterial communities not directly but indirectly by stressing the plant [51, 52]. Normal atmospheric concentrations of O_3_ already cause damage in crop plants [53]. NO_2_, in contrast, at normal atmospheric concentrations supports the plant by promoting nutrient uptake, plant growth, and metabolism [54]. Only artificial high amounts of NO_2_ would lead to negative effects on the plant [55]. Our data suggest that alterations of ambient air pollutants, whether favorable or unfavorable for the general growth condition, are accompanied by changes in bacterial composition colonizing the plant.

In general, higher NO_2_ and lower O_3_ concentrations are typically associated with high traffic urban areas. This gradient from rural to urban locations is distinctly shown by the significant positive correlation of NO_2_ and the significant negative correlation of O_3_ values to an increasing UI-value (S1 Fig). In detail, urban environments expose plants to a wide range of abiotic stressors, among them polluted soil, heat, drought stress and nutrient shortage [56]. All these factors, in case they occur in high intensity, can stress plants [19]. We did not analyze the influence of one of these additional stress factors to the plants. Therefore, we cannot suggest a direct relation of NO_2_ to the stress level in plants, because it could also be possible that another stress factor, which correlates positively with NO_2_ is the main stressor for the plants.

For measuring the stress level of the plants, we determined the activity of pollen NADPH oxidases. Besides the impact on allergic inflammation in the airways of animals and humans NADPH oxidases are involved in several processes of the plant system such as the response to biotic and/or abiotic stimuli (summarized in [57]). Our data show no correlation of NADPH oxidase activity to any of the measured pollution parameters. Neither was there any significant correlation of NADPH oxidase activity to microbial diversity. This suggests that NADPH oxidase activity in birch tree and timothy grass pollen is irrelevant for responses to abiotic stresses measured in this study.

A study analyzing the communities of dust-associated microbes found a declined microbial diversity in urban areas compared to rural areas [58]. We can confirm these investigations with regard to our results which show a declined bacterial diversity on birch pollen in urbanized areas. It is difficult to say what urban factors exactly might affect bacterial communities to the point of homogenization. There are studies showing high effects [59] and other studies showing little effect of urbanization to the atmospheric bacterial diversity [60]. Lower bacterial diversity might result from a higher homogenization of environmental conditions, land-use types or homogenized plant community in cities [58, 61]. Furthermore, there might be a link to the hypothesis that people living in urban areas often suffer from allergic diseases caused by an exposition to lower level of bacterial diversity [58].

### Correlation of Pollen Microbiome and Allergen/PALM Contents

In further correlation analyses, we investigated interrelationships between the microbial diversity and the pollen´s content of allergens and PALMs in birch tree and timothy grass pollen. Higher bacterial diversity (n(tRFs)) on birch pollen was found to be associated with higher concentrations of Bet v 1. Higher contents of PALMs were associated with a lower Shannon diversity on birch tree and timothy grass pollen.

Plants are able to recognize bacterial colonization via pattern recognition receptors (PRRs) on the plant cell surface [13, 14]. Microbe-recognition by PRRs induces a wide spectrum of defense responses in the plant [62]. The protein Bet v 1 belongs to the plant pathogenesis-related protein family 10 (PR-10). This protein is suggested to play a role in the response against pathogens or abiotic stress. In birch cell culture approaches it was detected that the gene expression encoding Bet v 1 isoforms is transcriptionally activated during pollen maturation and rapidly induced in the presence of microbes. Other stress factors like heat or heavy metals did not induce such an effect. An antibiotic function of enhanced Bet v 1 concentration seem to be likely, but tests with pollen washes including high amounts of Bet v 1 protein do not show any antibiotic effects [9]. In our study, we analyzed the interrelationship between microbes and Bet v 1 in natural habitats. In combination with the information we received from the cell culture approach explained above [9], the hypothesis of microbes influencing the expression of the allergen Bet v 1 might be supported. Future studies should address the effect of microbial species on the expression of Bet v 1 in pollen in more detail.

Finally, the diversity of low abundant bacteria on birch pollen was negatively correlated to the PALM content of pollen. Despite a low r_s_-value in case of PALM_PGE2_ the correlation was significant because of the high number of analyzed samples. The physiological roles of most PALMs are unknown to date. Some PALMs, e.g. the B_1_-Phytoprostanes are involved in the plant defense response against heavy metal and oxidants [11, 12]. High diversity of low abundance microbes, as measured by a high Shannon index, could reflect the presence of plant pathogens. In this case, PALMs production could be elevated in the course of pathogen-induced stress-responses. On the other hand, PALMs were measured by means of cross-reactivity to PGE_2_- and LTB_4_- antibodies in commercial ELISAs. It is therefore also possible that some lipids measured are not of pollen but of microbial origin. Detailed studies on pollen, from metabolome to function, will be required for dissecting the contribution of different PALMs *in planta* as well as their impact on the human immune system.

### Conclusion

In this study we characterized systematically bacterial and fungal communities colonizing allergenic pollen from birch tree and timothy grass. Summing up the gained results we showed that in pollen of different plant species significantly different microbial communities are represented. Moreover we found that the bacterial diversity on birch pollen decreases in more urbanized locations. Furthermore we were able to show a relationship between high bacterial diversity on pollen and the amounts of allergens and PALMs produced by pollen.

Based on our results and in accordance with other studies we suggest that pollution / urbanization influences the microbial load associated with pollen, which then probably impacts the expression of allergenic proteins and immune-modulatory and stimulatory PALMs in the plant pollen. Up to now, the bacterial colonization of pollen was treated as a factor stressing the plant. We point out that the well-known and important beneficial and symbiotic effects of bacteria must not be neglected, too. With regard to the increasing number of incidences of allergic diseases, it is necessary to reveal to what extent anthropogenic factors influence the allergenic potential of pollen. Further studies should shed light on the molecular interplay on and in pollen grains. These studies should also clear whether there are certain bacterial isolates that might harbor key-functions in terms of modulating the expression of immune-related compounds.

## Supporting Information

S1 Fig Correlation of pollution parameters NO_2_ and O_3_ to the Urbanization Index (UI). Ambient NO_2_ concetration is significantly positive correlated to the Urbanization Index (UI; rho = 0.68, p < 0.0001), whereas ambient O_3_ concentration is significantly negative correlated to the Urbanization Index (rho = -0.46, p < 0.001).
(TIF)

S1 Table Correlation of fungal diversity on birch pollen to the content of PALMs and allergens, to the activity of NADPH oxidase as well as to measured pollution parameters. Spearman-Correlation of fungal diversity-indices (Simpson, Shannon) and the absolute number of different fragments (n(tRFs)) analyzed from birch pollen (*Betula pendula*, 2014) to air pollution concentration (NO_2_, O_3_, NH_3_; n = 31), the produced amount of allergens and PALMs (bet v 1, PALM_PGE2_, PALM_LTB4_, n = 31) and the activity of NADPH oxidase (n = 18). p = significance level.
(PDF)

S2 Table Correlation of bacterial diversity on timothy grass pollen to the content of PALMs and allergens and to the Urbanization Index (UI). Spearman-Correlation of bacterial diversity-indices (Simpson, Shannon) and the absolute number of different fragments (n(tRFs)) analyzed from timothy grass pollen (*Phleum pratense*, n = 20; 2014) to the produced amount of allergens (Phl p 5), the amount of PALMs (PALM_PGE2_, PALM_LTB4_) and the Urbanization Index (UI). p = significance level.
(PDF)

S3 Table Correlation of fungal diversity on timothy grass pollen to the content of PALMs and allergens and to the Urbanization Index (UI). Spearman-Correlation of fungal diversity-indices (Simpson, Shannon) and the absolute number of different fragments (n(tRFs)) analyzed from timothy grass pollen (*Phleum pratense*, 2014) to the produced amount of allergens (Phl p 5; n = 20) and PALMs (PALM_PGE2_, PALM_LTB4_, n = 20) and also to the Urbanization Index (UI; n = 18). p = significance level.
(PDF)
