# Supplementary figures and images for: Pollen-Associated Microbiome Correlates with Pollution Parameters and the Allergenicity of Pollen
Source: PLoS One. 2016 Feb 24;11(2):e0149545. doi: 10.1371/journal.pone.0149545 (PMC4765992; doi:10.1371/journal.pone.0149545)

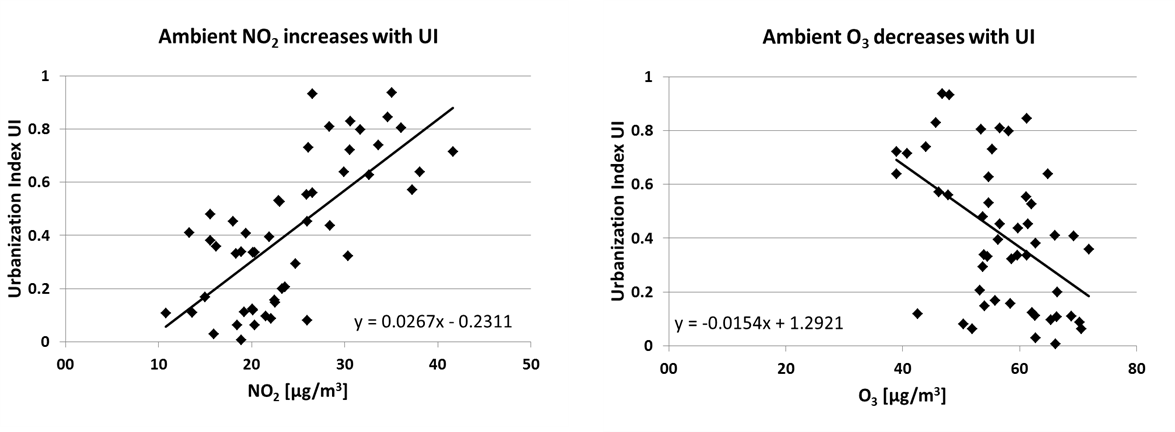

Supplement: S1 Fig — Ambient NO2 concetration is significantly positive correlated to the Urbanization Index (UI; rho = 0.68, p < 0.0001), whereas ambient O3 concentration is significantly negative correlated to the Urbanization Index (rho = -0.46, p < 0.001). (TIF) [file pone.0149545.s001.tif]
