# Supplementary material for: Pollen-Associated Microbiome Correlates with Pollution Parameters and the Allergenicity of Pollen
Source: PLoS One. 2016 Feb 24;11(2):e0149545. doi: 10.1371/journal.pone.0149545 (PMC4765992; doi:10.1371/journal.pone.0149545)
Supplement: S1 Table — Spearman-Correlation of fungal diversity-indices (Simpson, Shannon) and the absolute number of different fragments (n(tRFs)) analyzed from birch pollen (Betula pendula, 2014) to air pollution concentration (NO2, O3, NH3; n = 31), the produced amount of allergens and PALMs (bet v 1, PALMPGE2, PALMLTB4, n = 31) and the activity of NADPH oxidase (n = 18). p = significance level. (PDF) [file pone.0149545.s002.pdf]

	pollution			allergenicity			Physiol.
**Diversity-Index**	**NO_2 [µg/m^3]**	**O_3 [µg/m^3]**	**NH_3 [µg/m^3]**	**Bet v 1 [ng/10ml]**	**$PALM_{PGE2}$ [pg/ml]**	**$PALM_{LTB4}$ [pg/ml]**	**NADPH oxidase**
**Simp. 1-D**	p = 0.53	p = 0.86	p = 0.95	p = 0.69	p = 0.61	p = 0.21	p = 0.96
**Shannon H**	p = 0.54	p = 0.65	p = 0.95	p = 0.64	p = 0.58	p = 0.15	p = 0.82
**n(tRFs)**	p = 0.79	p = 0.39	p = 0.98	p = 0.69	p = 0.36	p = 0.13	p = 0.55
