# Supplementary material for: Pollen-Associated Microbiome Correlates with Pollution Parameters and the Allergenicity of Pollen
Source: PLoS One. 2016 Feb 24;11(2):e0149545. doi: 10.1371/journal.pone.0149545 (PMC4765992; doi:10.1371/journal.pone.0149545)
Supplement: S2 Table — Spearman-Correlation of bacterial diversity-indices (Simpson, Shannon) and the absolute number of different fragments (n(tRFs)) analyzed from timothy grass pollen (Phleum pratense, n = 20; 2014) to the produced amount of allergens (Phl p 5), the amount of PALMs (PALMPGE2, PALMLTB4) and the Urbanization Index (UI). p = significance level. (PDF) [file pone.0149545.s003.pdf]

Diversitäts-Index	Phl p5 [pg/ml]	$PALM_{PGE2}$ [pg/ml]	$PALM_{LTB4}$ [pg/ml]	Urbanization Index
**Simpson 1-D**	p = 0.70	**p = 0.039**	p = 0.06	**p = 0.04**
**Shannon H**	p = 0.46	p = 0.57	p = 0.67	p = 0.14
**n(tRFs)**	p = 0.25	p = 0.26	p = 0.10	**p = 0.04**
