# Supplementary material for: Pollen-Associated Microbiome Correlates with Pollution Parameters and the Allergenicity of Pollen
Source: PLoS One. 2016 Feb 24;11(2):e0149545. doi: 10.1371/journal.pone.0149545 (PMC4765992; doi:10.1371/journal.pone.0149545)
Supplement: S3 Table — Spearman-Correlation of fungal diversity-indices (Simpson, Shannon) and the absolute number of different fragments (n(tRFs)) analyzed from timothy grass pollen (Phleum pratense, 2014) to the produced amount of allergens (Phl p 5; n = 20) and PALMs (PALMPGE2, PALMLTB4, n = 20) and also to the Urbanization Index (UI; n = 18). p = significance level. (PDF) [file pone.0149545.s004.pdf]

**Diversitäts-Index**	**Phl p5 [pg/ml]**	**$PALM_{PGE2}$ [pg/ml]**	**$PALM_{LTB4}$ [pg/ml]**	**Urbanization Index**
**Simpson 1-D**	p = 0.64	p = 0.81	p = 0.09	p = 0.20
**Shannon H**	p = 0.64	p = 0.92	p = 0.21	p = 0.32
**N(tRFs)**	p = 0.35	p = 0.85	p = 0.84	p = 0.63
